# Chicago Gynæcological Society

**Published:** 1888-01

**Authors:** 


### CHICAGO GYNÆCOLOGICAL SOCIETY TRANSACTIONS.

REGULAR MEETING, SEPTEMBER 24, 1887.

The President, Charles Warrington Earle, M. D., in the Chair.

Professor J. H. Etheridge read the following paper, entitled, *Vaginal Hysterectomy*; report of three cases operated on at the Presbyterian Hospital. He said: "I can never convey an adequate idea of the relief to the operator offered by the method of hemostasis by forcipressure on the broad ligaments, as compared with that of ligatures. I think no one can fully appreciate the untold superiority of the former method over the latter till he has had experience in the performance of that operation under both methods."

CASE I. Mrs. S., æt. 47, mother of nine children. She had otherwise always been well. She presented herself February 1, 1887, with epithelioma of the cervix uteri. It did not involve the vault of the vaginia. The broad ligaments did not seem to be thickened. Mobility of the uterus was complete. After preparatory treatment with a daily laxative and diuretic for one week, the operation was performed on February 8, 1887.

The cervix was easily drawn down to the vulvar orifice, and with scissors its vaginal attachment was divided. Strong adhesions to the bladder and rectum were found, and, in consequence thereof, the rectum was opened in one place and the bladder in two places in the process of freeing the uterus from these two organs. After the two broad ligaments were sufficiently isolated, and the fundus was turned backwards and brought down, the left broad ligament was first penetrated and divided into two sections with broad ligatures, and tied as securely as hands could tie them. It was then severed as close to the corpus uteri as was possible, and the whole organ came out of the vagina. Treating the right broad ligament similarly was a much easier matter, because the uterus was out of the way. This attachment was at once severed, and the whole organ was then from the patient. The ovaries were subsequently removed.

The rent in the bowel was closed by continuous suture without difficulty. The larger rent in the bladder was also closed by continuous suture; but it was done at a great disadvantage, from its peculiar position behind the symphysis pubis and looking directly backwards. To draw down the bladder and to so evert the edges of the rent as to apply the stitches was a delicate and difficult task. The smaller rent, undiscovered at that time, was not closed up. Just as this sewing up was completed, there was observed welling up into the shapeless excavation left after the removal of the uterus great quantities of arterial blood. From which broad ligament it came it was impossible to decide. After a long time the bleeding vessel, which was in the right broad ligament, was secured, but not till after a ligature had been pushed off the left broad ligament. All vessels were eventually secured, but not till a great quantity of blood had been lost.

The top of the vagina was closed from before backwards with a continuous suture, the ligatures were brought down, iodoform-gauze stuffed into the vagina, and the patient put to bed. Reaction followed, but slowly. She died from peritonitis and exhaustion in forty-five hours, having passed eight ounces and one drachm of urine in the meantime.

The autopsy revealed a small rent in the bladder, which it was concluded had been the cause of the peritonitis.

CASE II. Mrs. C., æt. 36 years, tall, spare, nervous, and of sanguine temperament, presented herself February 10, 1887, with a small epithelioma of the cervix uteri. The upper portion of the vagina cervix was not involved. The choice between amputation of the cervix and hysterectomy was left to the patient.

After full explanation of the danger and results of the two procedures, she decided to have the latter operation, which was performed on February 25, 1887.

The uterus was easily drawn down to the vulvar orifice and freed from its vaginal attachments by the use of scissors. The bladder was uncommonly closely attached to the uterus, and before its complete separation was accomplished, it was opened. The opening into the Douglas cul-de-sac was easily effected, and the fundus turned backward through the sacral hollow, down and out through the vulva. The broad ligaments were secured with silk ligatures, and the uterus removed after its separation from them. The ovaries were separately removed immediately afterwards. In closing the vesical rent, the ligatures slipped the left broad ligament and it bled profusely. The hæmorrhage was soon checked and the vagina was closed from before backwards. The ligatures were brought down into the vagina, and the latter organ was filled with iodoform - gauze. The patient rallied well. The temperature rose to 100° F. on the second and third days. Thereafter nothing worthy of special mention occurred. On the tenth day, an elastic ligature, attached to the patient's left thigh, was tied to those protruding from the vagina, and in five days they began to come away, and in forty-eight hours afterwards the last one was removed. In thirty-six days she left the hospital.

CASE III. Mrs. C., æt. 49, a widow, appeared on April 13, 1887. Her last confinement had been twenty-eight years before. She was still menstruating regularly every three weeks, flowing one week each time.

Six months before, she began to have leucorrhœa and to lose occasional small amounts of blood. She had excellent general health. Her appetite and digestion were good. Her bowels, kidneys, and skin acted naturally. She was well nourished and presented a promising prospect for any surgical ordeal. One thing that one could wish different was a too rapidly acting heart. It beat over ninety times a minute, and the arterial impulse was persistent. She had often seen lateritious deposits in her renal secretion.

Examination revealed an epitheliomatous degeneration of the cervix, with about one-fourth of an inch of uninvaded tissue of the cervix between the cancer and the vaginal vault. The uterus was about four inches deep, and it bled freely upon withdrawal of the sound. The fundus was large, and was easily felt through the abdominal wall. The uterus was freely movable, indicating the non-implication of the lymphatics in the broad ligament. The absence of invasion of the vaginal wall and of the circum-uterine tissues led to the recommendation of an operation for removal of the whole uterus.

From April 19 to May 5, the date of the operation, she took cascara daily, and digitalis and kalium acetate. The condition of the excretions seemed to be as nearly perfect as possible preparatory to an operation. She slept in the hospital the night before the operation, and took the customary antiseptic general bath, and had administered several vaginal bichloride douches.

*The operation.*—The cervix was drawn into the vulva with two large, locked vulsella forceps, while the vaginal attachment to the cervix was divided with scissors. Gradually and patiently the circumcervical tissues and the attachments of the bladder and rectum were crowded away with the finger nail till the Douglas cul-de-sac could be opened. Then it was found quite impossible to reach the top of the fundus with the fingers. The cul-de-sac of peritoneum between the bladder and the uterus was then opened, with the hope of being able to retroflex the uterus by means of the fingers placed before and behind it. This manœuvre was found to be an impossibility. After repeated vain attempts to reach the top of the fundus with the fingers that method was abandoned. Trial of very deep supra-pubic pressure to thrust the fundus back towards the sacral hollow, and at the same time to grasp and pull down the fundus with a small vulsella forceps thrust through the Douglas cul-de-sac, at last succeeded in getting the top of the uterus out of the vagina, but only after the forceps had torn loose several times. Snap forceps were then placed on the broad ligaments and the latter divided. The subsequent dressing consisted in tucking a thin layer of iodoform-gauze into the vagina, care being taken to avoid separating the top of the vaginal walls. The danger of this separation must be evident to any observer. Ribollet attributes the death of one of his patients to crowding too much gauze into the upper vagina.

No stitches were used to close the upper end of the vagina. Its borders were permitted to collapse and to close in any position that they chanced to occupy. Fear that the bowels and bladder might seek an outlet through the vaginal tract is wholly groundless. One ligature was used for a vaginal artery. No attention was paid to it in the final dressing.

The ovaries were both removed after the uterus was finally separated from its attachments.

The patient reacted well from the shock of the operation, which consumed seventy-five minutes. Her daily progress was so uniformly satisfactory that any detailed descriptive statement of it would be unnecessary.

The pulse ranged from 90 to 120 beats per minute. It was 98 when she left the hospital. The temperature reached 100°F. for one morning only, and on five evenings, from the third to the seventh days, inclusive.

The amount of urine passed daily during the first fourteen days after the operation is indicated by ounces in the following figures: 19, 19½, 25, 33, 26, 24, 23½, 25, 30, 31½, 34, 40, 35, and 26½.

The forceps, one pair on each broad ligament, were removed at the end of forty-eight hours. Although one knows that forty-eight hours of *closure* of the lumen of an artery will necessarily destroy its patency, yet the writer had some misgiving when the forceps were very carefully unsnapped and carefully removed. No bleeding followed the removal of pressure from the broad ligaments. The vagina was not filled with gauze. Iodoform was daily blown into it as far as was possible.

It was impossible to state definitely the amount of drainage that escaped; perhaps three tablespoonfuls daily, for the first two days, would cover it all; afterwards the amount could not have been more than one tablespoonful on the third and fourth days each.

The following are some points of interest concerning this operation.

*Indications for its performance.*—(*a*) Ten years ago, and, indeed, until quite recently, the chief indication for the performance of vaginal hysterectomy was malignant disease. At present it is agreed by all operators that the earlier it is performed for cancer the greater are the chances for its non-recurrence. This dreaded malady always returns sooner or later after amputation of the uterine cervix, and of course proves fatal; whereas, when the whole organ is removed, the patient is given the only hope of permanent recovery. Hysterectomy does not always prevent recurrence of the development of this neoplasm, yet it offers the best results. Martin reports eight cases of hysterectomy for cancer, without relapse, varying from two and one half to five years. According to Sänger, the average of survival after this operation is eleven months. Olshausen reports cases, after operation, of relapse, once after eighteen months, twice after two years, and twice after three years. Seven patients had survived two years without recurrence, and seven other women had lived over one year without relapse. Recurrence usually takes place in the seventh month after removal of cancer, and sixteen cases of F. would seem from his report to be in a fair way for complete recovery.

The most favorable conditions offered for hysterectomy are the non-involvement of the vagina, and complete mobilization of the uterus, which shows the non-involvement of the *ligamenta lata*. In other words, the earlier in the disease the operation can be performed, the better are the promises of a radical cure. Frequently it occurs that the disease has advanced too far before the gynæcologist is consulted.

(*b*) *Procidentia uteri* is another condition for which this operation is performed. Anaplastic operations do not always restore the organ to its normal level. Artificial vaginal stenosis to the extent of the non-admission of the little finger has failed ultimately to relieve the procidentia through gradual dilatation of the vaginal channel.

(*c*) Fibrous bodies of the uterus which offer the point of departure of serious irregularities, have constituted a cause for vaginal hysterectomy. Of course reference is had to small tumors. Hedenreich* reports four cases of operation with four successes. He considers that at present it is impossible to pronounce upon the relative merits of vaginal hysterectomy and of castration for small fibrous bodies in the uterus. Péan † recently reported a case of the same operation for multiple fibroids.

(*d*) The hystero-neuroses (inveterate dysmenorrhœa, neuralgia, convulsion, etc.), for which oöphorectomy is so often performed, Péan considers a justifiable cause for this surgical procedure. His reasoning is that these neuroses sustain an intimate relation to the uterus itself, consequently the uterus should be included along with the tubes and ovaries. (Caldwell, Paris letter in Chicago *Medical Journal and Examiner*, February, 1887.)

As an illustration of the *furor operativus*, a recent article from the pen of a Cologne surgeon, Dr. Frank, may be mentioned, which was published in the April 3, 1887, number of the *Archiv. für Gynäkologie*, in which are enumerated the following cases of removal of the entire uterus:

For endometritis, four cases; for retroflexion or retroversion with fixation, three cases; pruritus uterinus, one case, and for neuralgia and retension of urine, one case.

Members of the medical profession can scarcely read the account of these cases without being astounded at the amazing temerity of such proceedings.

The various steps of the operation consist in (1) freeing the cervix from its attachments, (2) hemostasis, and (3) the subsequent dressing.

Professor Christian Fenger exhibited specimens of *Carcinoma of the Cervix*.

The first specimen was from a woman about 40 years of age, a multipara. She had symptoms of the affection for over a year, and was in rather an emaciated condition, partly on account of chronic bronchitis and a cystic goitre, and partly on account of the carcinoma, where several local operations had been performed before. It was a cervix carcinoma, with the cervix involved almost to the internal os, the white mass below being the carcinoma tissue, and all of the rest of the cervix being carcinomatous. He was induced to operate in the case by the extreme mobility of the uterus. As a rule, in carcinomas that have gone as far as the specimen exhibited, operation is not advisable; it is too late. However, she was not asked to be operated upon, but she implored him to operate on her at any risk to her life, and he operated. Before the operation her pulse was 120, and she was, as stated be-

* Hedenreich, Albert. "De hysterectomie vaginale," (Semaine Med., Paris, 1886, vi., 69-70).

† Gaz. des Hopitaux, October 12, 1886, pp. 950-951.

fore, weak, but the uterus was movable and was taken out without any considerable difficulty. Before the end of the operation, which lasted about two hours, she was very weak and almost pulseless. This condition lasted after the operation, and in the evening her pulse was 170 and could scarcely be counted. She did not lose any quantity of blood to speak of. He made a saline infusion of twelve or thirteen ounces in the brachial vein, and afterwards the pulse got stronger, and continued so from that time, and there was no further trouble during her recovery.

The next specimen was a portio carcinoma extending about an inch into the cervix on the posterior lip. It was a portio carcinoma because it had its greatest extent down at the vaginal portion, and became smaller and smaller as it went up in the cervix. In a cervix carcinoma that opens down in the vaginal portion, we should expect to have a larger cavity in the cervix and a smaller opening in the vaginal portion. This patient was 28 years old, and in spite of her being a nullipara the operation was very easy. As in all of the cases, it was necessary to dilate the vagina posteriorly, but otherwise, evidently on account of the small size of the uterus, the operation was easy—so easy that there was no cause for either ante- or retro-version while taking it out; it was just held down, and the ligaments ligated. In this case the operation was so easy that he united the peritoneal as well as the vaginal wound, closed them up together with a row of sutures, commencing in the anterior fornix, going through the anterior peritoneum, the posterior peritoneum, and out of the posterior fornix, thus closing up by one row of sutures the peritoneal and the vaginal wound. In the other case, he closed the peritoneal wound and left the vaginal wound open. The drainage used was iodoform gauze. She made the most undisturbed recovery of all of them; never had a rise of temperature nor of pulse, and never had any pain. The tubes and ovaries were not removed.

The third case was a small portio carcinoma, extending but slightly up in the cervix, and could be seen on the posterior surface of the uterus. She had previously had perimetritis. This uterus was perfectly movable, but the operation was not easy, as the loosening of the bladder from the uterus was difficult, while in the first two cases it was easily detached with the finger. Perhaps on account of the previous perimetritis, the tissue between the bladder and uterus was so dense that it had to be cut with the scissors, and he had made a small opening into the bladder which was united at the time it was cut, and did not leave any bad results, not even a temporary fistula, as no urine passed at any time down into the vagina. Her recovery was uninterrupted.

The fourth specimen was another portio carcinoma on the posterior lip, reaching up in the cervix perhaps half an inch. The patient was a multipara, 28 years old, and quite fleshy. Contrary to expectation, the operation was extremely difficult. The vagina was not unusually narrow, but the difficulty of the operation was due partly to the large size of the uterus, and partly on account of the fact that she was very fleshy. In a very fleshy person there is some little part of the vagina narrowed by the subcutaneous tissue on the inside of the nates. There was in this case the same difficulty as in the previous one about separating the bladder from the uterus, but it did not cause any passage of urine into the vagina. He operated according to the method of Leopold, loosening the lower part of the broad ligaments first, and then going up until there are no firm bands left on the sides of the uterus. When that is done, and the anterior fornix loosened, as a rule the uterus becomes so movable that it comes down an inch or an inch and a half or two inches into the vulva, so that the rest of the operation is comparatively easy. But in this case it did not come down. There was an unyielding condition of the lateral ligaments.

The reason for it was not known. This condition made the operation difficult and long. At the end of the operation it was thought best not to lose time in uniting the peritoneal or the vaginal wound. He only brought down the ligatures to the border of the vaginal incision, uniting them there and leaving the peritoneal cavity open, using for drainage iodoform-gauze packed up in the peritoneal cavity. This patient did not have an uninterrupted recovery. She had a temperature for a couple of days of about 100.5°F., later on, 100°, later, below a hundred. What was more alarming than the temperature was that the pulse was up in the neighborhood of 120 most of the time. In this case he had to change the gauze packing at the beginning of the rise in temperature, taking it out and introducing a drainage tube so as to be able to wash it out every day. The washing-out, however, was not begun until after the tenth day, because, at least one, perhaps more cases are on record where, when the washing-out was performed early, the fluid had gone up into the peritoneal cavity and caused general infection.

*Dr. Merriman:* Do you think that if you had sewed up the cavity of the peritoneum you would have saved her much?

*Professor Fenger:* I must confess that I believe in closing up the peritoneal wound as a matter of safety.

These specimens show one point, namely, that a strict line, as Ruge and Veit have pointed out, between portio carcinomas and cervix carcinomas does not exist. They say that a portio carcinoma very rarely extends up into the cervix. If that is the case, it would, of course, not be justifiable in many cases of simple portio carcinoma to do anything but the partial operation. Fritsch has called attention to a fact which these specimens show very distinctly—that is, the difficulty of finding out if a portio carcinoma is limited to the portio proper, or extends up into the cervix or even into the cavity of the corpus.

I might say a few words in regard to the manner of operating. It is very far from being generally agreed upon which operation is the best, and the variety of procedures is very large. As an illustration, we will take the treatment of the broad ligament and the closing of the vaginal and peritoneal wound. I think there has been an improvement in the technique of operating over the old methods in operating as proposed by Sänger and Fritsch. They begin the operation by the ligaturing step by step of the parametria. When that is done well on both sides, the uterus will become so movable that the remainder of the operation can be done with comparative ease. This ligaturing step by step, together with Martin's method of suturing, does away with one of the dangers of the operation, namely, hæmorrhage. By this step ligaturing, the uterine artery is met half or three-quarters of an inch above the fornix, and can be securely ligated so that the rest of the operation can be done with comparatively little loss of blood. The other danger which we have to encounter is sepsis, as we have to do with a carcinoma whose surface is always decomposed and septic. Besides doing the usual and necessary clean operating, Leopold proposes that we should not antevert or retrovert the uterus, but take it out without any of these procedures. Fritsch advises to leave the posterior fornix closed when we antevert, so that the carcinomatous surface cannot be turned up into the peritoneal cavity and come in contact with the organs there.

As to the treatment of the peritoneal and vaginal wound, there are also a great variety of methods. It seems that for after-treatment packing with iodoform gauze is much more convenient and much less troublesome than a drainage tube, as the iodoform gauze can be left in for a week or more without being removed, and then the after-treatment is over.

*Dr. Nelson:* I would like to ask Dr. Fenger in regard to the removal of the ovaries and tubes, whether they are likely to produce disagreeable results in the after-history of the patient by being retained. I can understand full well the desirability of leaving as many organs and as much tissue as can be left, but would raise the question of the desirability or not of removal of the ovaries and tubes when the uterus has been removed.

*Professor Fenger:* In answer to that question, I would say that it is remarkable what little trouble has been reported from the cases where the ovaries and tubes have been left. There are a few cases, one of Schroeder's, where the menstrual molimina from the organs left has caused the patient trouble. In another case the ovary, or a piece of it, which had been left, was imbedded in the cicatrix, and caused afterwards, according to the opinion of the operator, periodical pains. Brennecke says that he has come to the conclusion that it does not do any harm to leave the ovaries and tubes in. He says that the ovaries atrophy; that some of his patients have had slight molimina in the first three to six or nine months after the operation, and after that time the symptoms from the ovaries have always entirely ceased. That is all I know about this question. But as we get towards the end of the operation it is hard on the patient and hard on the operator, and I feel like doing as little additional operating as possible, and if I can leave the ovaries and tubes without doing the patient any harm it is preferable to do so, as the operation in many cases is a severe one on account of the liability of the patient to collapse towards the end of the operation, which lasts for an hour and a half to two hours. So until I can do the operation much quicker I should prefer to leave the ovaries and tubes in.

*Dr. Merriman:* Is there any danger of including the ureter in this operation, of injuring it in any way?

*Professor Fenger:* Yes; there is danger, inasmuch as it has been done, although very rarely. When the ligaturing of the broad ligaments has been done step by step, so that the uterus can be drawn down, then the ureter stays up, so that the final ligature of the broad ligaments is not likely to include the ureter.

*Dr. Merriman:* He draws the bladder down with it, the uterus and bladder not being disconnected, and I should think there would be great danger of including the ureter in some of the operations.

*Professor Fenger:* Even if the ureter is caught with the bladder when the uterus is made movable and drawn down, the ureter stays up.

*Dr. Nelson:* Is it customary with the majority of operators to curette and to thoroughly disinfect the carcinomatous ulcer before beginning the operation, immediately before, or is it done several days before, or is it done at all?

*Professor Fenger:* I think it is done several days before by some; for instance, by Hegar and some others. Fritsch and Leopold do it at the beginning of the operation. After the curetting, the surface is disinfected by a strong solution of chloride of zinc or a strong carbolic-acid solution. I prefer to postpone it to the beginning of the operation, so as not to have the patient disturbed with an additional operation, with some loss of blood.

*Dr. Merriman:* Supposing there was a case of cancer that had gone beyond the cervix into the tissue posterior to the uterus, invading the vagina and extending along down on the anterior side, but there was nothing anterior to the uterus, would it be safe, in a case of that kind, to undertake the operation of extirpation?

*Professor Fenger:* I think I should refuse. I think the benefit to the patient lies in operating early; and as soon as a portio carcinoma has gone over onto the wall of the vagina to any extent I think the prospects of a radical cure are very small, and that the patient is just as well off with symptomatic treatment, curetting, etc. I think the aim of the operation should be a radical cure, and that extensive operating is being done away with more and more, and only limited cases regarded fit for operation.

It is generally accepted as a law in the surgery of the mammary gland that no matter how localized a small carcinomatous nodule may be, nothing less than the removal of the entire gland, and I would regard it safe to add, the lymphatics of the axilla, would be the safe operative procedure to adopt. It can thus be understood that authors who believe in a low mortality for the vaginal hysterectomy — Sänger, Leopold, Fritsch — require this operation to be done in all cases of limited carcinomas, even small carcinoma of the vaginal portion, to the exclusion of any of the partial operations.

Fritsch calls attention to the fact that a strict demarkation line between carcinoma of the cervix and of the vaginal portion, as Ruge and Veit in their classical article on uterine carcinomas have described it, does not exist in all cases. Some apparent portio carcinomas may extend deeply up into the cervix. It is often not possible during a partial operation, viz., vaginal amputation of the cervix, to determine if we amputate in healthy tissue.

Consequently, for the majority of such cases, the total extirpation is safer as to the radical cure of the carcinoma than a partial operation.

*Dr. H. T. Byford:* I had the good fortune to successfully operate upon one case in which the uterus looked something like this larger one Professor Fenger has shown us. It was probably an inch longer. The patient was a fleshy, married lady, about thirty years old. The cervix had been amputated a few months before. I ligated the broad ligaments step by step, as recommended by Leopold. I left the abdominal cavity open, except to draw the ligatured parts together, and then packed the vagina with iodoform-gauze for eight days. By mistake one of the iodoform-tampons was left in nearly two weeks, but it did not cause any serious symptoms. As to leaving the peritoneal cavity open, I think there will never be any fixed rule, for that will probably have to be decided by the case. If the case was one in which there was no such manipulation of the broad ligaments, we would expect some sero-sanguineous exudation, and should not completely close the peritoneal cavity. When we use iodoform-gauze in the proper shape we practically close it up. I do not see the use of sutures. Before there can be any exudation of secretions the peritoneal cavity is closed by exudation. Stitches are only a source of irritation both during and after being inserted. As to leaving the ovaries, it seems to me that in taking out the uterus we take out the larger portion of the sexual nervous system, and produce atrophy of the sexual organs quicker by removing the uterus than by removing the ovaries. It would be almost as superfluous to remove the ovaries after taking out the uterus as to remove the uterus after taking out the ovaries to bring on the menopause. There is one kind of operation that has not been referred to—that is, by leaving compression forceps on the stumps, as is done quite extensively in France and England. When the patient is very weak, it seems to me an improvement to put on these clamps and thus rapidly finish the operation. In regard to the mortality and difficulty of the operation, I think that taking out the uterus is not so very much more serious, though much more difficult, than amputating the cervix. It can not be so safely done so often by the experienced operator, for he is more liable to do something that will endanger the life of his patient. But when the operation is properly performed, the peritoneal cavity being practically closed by the parts being brought together, the patient is left in as good condition for recovery as by a high amputation combined with the cautery.

*Dr. J. C. Hoag:* I have observed with a good deal of interest an apparent revulsion in feeling and opinion in regard to the advisability of this operation. Only a few months ago, in conversation with a number of operators in England, I found the operation was very generally decried, but since that time, from a perusal of the English journals, one gets a different idea of the opinions of British operators. Some of them did not hesitate to say that in those cases which recovered there was no carcinoma.

In regard to the technique of the operation, I can only speak in the light of what experience I have had in operations on the cadaver, under the instruction of a prominent operator. I was instructed to begin the operation by opening the anterior and posterior culs-de-sac. This certainly seems to be an inferior method to the one described by Dr. Fenger, as affording opportunity for infection of the peritoneum. It has one advantage, however, and that is, it enables one to get his bearings better in regard to the relations of the parts, because one can surround the broad ligaments with the finger and find just where to pass the ligatures, and in this way I think the ligatures can be placed in a more accurate manner. The loss of blood is less, and after the removal of the uterus there is no trouble from hæmorrhage, because it is *entirely* prevented by the accurately applied ligatures, whereas in the other methods there is often a considerable loss of blood. In a number of cases which I saw, the operation was practically bloodless.

He related the following history of a case of *placenta prœvia.*

He said : I wish to refer to a case which I attended recently in which there was one point of interest; it was a case of placenta prævia which I saw a few days ago in consultation. I was called early in the morning, and on going to the patient found her in labor, the labor being a little premature by perhaps three or four weeks. She had suffered repeatedly from severe loss of blood during the last three months. At the time I saw her the os was sufficiently dilated to admit one finger only; the periphery of the placenta could be felt throughout perhaps a fourth of its extent. She was having no particular hæmorrhage at that time, but had been flowing all night. I endeavored a few hours later to introduce a Barnes dilator, but failed because the cervix was very unfavorably situated, being so far back in the cervix that it was impossible to introduce even a small dilator. I introduced a colpeurynter and left it for three or four hours, by which time the os was found to be pretty well dilated, and the remainder of the management was left to the other physician at his request, as he had not previously attended one of these cases. By external manipulation, palpation and auscultation, I had no difficulty in finding the exact location of the feet; the other physician introduced his hand and with little effort was able to pass it into the uterus; he seized the foot through the membranes, but had great difficulty in holding it. There was a good deal of difficulty in rupturing the membranes. I tried it before the introduction of the colpeurynter, but gave it up and advised the physician to rupture the membranes wherever he could do so. He soon succeeded in doing this, and the case offered no difficulties afterward. There was very little loss of blood. The patient was afterwards given two antiseptic douches per day, and has done very well since. The child is living.

DISCUSSION.

*Dr. Merriman :* How much hæmorrhage was there after you introduced the colpeurynter?

*Dr. Hoag :* Very little.

*Professor Earle :* Did she get an intrauterine douche immediately after the operation ?

*Dr. Hoag :* Yes, immediately; before the colpeurynter was put in and after the delivery of the child, and she has had no temperature above 99° F.

